# Co-expression of SpSOS1 and SpAHA1 in transgenic *Arabidopsis* plants improves salinity tolerance

**DOI:** 10.1186/s12870-019-1680-7

**Published:** 2019-02-14

**Authors:** Yafei Fan, Xiaochang Yin, Qing Xie, Youquan Xia, Zhenyu Wang, Jie Song, Yang Zhou, Xingyu Jiang

**Affiliations:** 1 Hainan Key Laboratory for Sustainable Utilization of Tropical Bioresources /Institute of Tropical Agriculture and Forestry, Hainan University, Haikou, 570228 China; 2 Shandong Key Laboratory of Plant Stress/College of Life Science, Shandong Normal University, Jinan, 250014 China

**Keywords:** H^+^-ATPase, Na^+^/H^+^ antiporter, Plasma membrane, Salt tolerance, *Sesuvium portulacastrum*

## Abstract

**Background:**

Na^+^ extrusion from cells is important for plant growth in high saline environments. SOS1 (salt overly sensitive 1), an Na^+^/H^+^ antiporter located in the plasma membrane (PM), functions in toxic Na^+^ extrusion from cells using energy from an electrochemical proton gradient produced by a PM-localized H^+^-ATPase (AHA). Therefore, SOS1 and AHA are involved in plant adaption to salt stress.

**Results:**

In this study, the genes encoding SOS1 and AHA from the halophyte *Sesuvium portulacastrum* (*SpSOS1* and *SpAHA1*, respectively) were introduced together or singly into *Arabidopsis* plants. The results indicated that either SpSOS1 or SpAHA1 conferred salt tolerance to transgenic plants and, as expected, *Arabidopsis* plants expressing both *SpSOS1* and *SpAHA1* grew better under salt stress than plants expressing only *SpSOS1* or *SpAHA1*. In response to NaCl treatment, Na^+^ and H^+^ in the roots of plants transformed with *SpSOS1* or *SpAHA1* effluxed faster than wild-type (WT) plant roots. Furthermore, roots co-expressing *SpSOS1* and *SpAHA1* had higher Na^+^ and H^+^ efflux rates than single *SpSOS1*/*SpAHA1-*expressing transgenic plants, resulting in the former amassing less Na^+^ than the latter. As seen from comparative analyses of plants exposed to salinity stress, the malondialdehyde (MDA) content was lowest in the co-transgenic *SpSOS1* and *SpAHA1* plants, but the K^+^ level was the highest.

**Conclusion:**

These results suggest SpSOS1 and SpAHA1 coordinate to alleviate salt toxicity by increasing the efficiency of Na^+^ extrusion to maintain K^+^ homeostasis and protect the PM from oxidative damage induced by salt stress.

**Electronic supplementary material:**

The online version of this article (10.1186/s12870-019-1680-7) contains supplementary material, which is available to authorized users.

## Background

Salts, particularly NaCl, can be toxic to plants through inhibition of important biochemical and physiological processes, such as protein synthesis, photosynthesis, and enzymatic reactions, after moving into the cytosol from soils [1]. While salt stress can inhibit plant growth and development, many types of plants are able to grow in high salinity environments because they have complex mechanisms that facilitate adaptation to salinity stress [2]. Of these mechanisms, the ability to transport excess Na^+^ out of cells is critical to salt tolerance. SOS1 (salt overly sensitive 1) is a Na^+^/H^+^ antiporter and the only Na^+^ efflux protein present in plant plasma membranes (PMs) characterized to date. SOS1 mediates extrusion of Na^+^ through a proton gradient generated by the H^+^-ATPase (AHA) in the PM [3]. Therefore, SOS1 and AHA are two key plant halotolerance factors.

PM H^+^-ATPase is encoded by a large family of genes [4, 5]. Bioinformatics analyses of *Arabidopsi*s and genomic sequences of rice revealed the presence of 11 and 10 PM AHAs, respectively [6, 7]. Of these AHAs, NaCl treatment induced expression of three*, AtAHA1, AtAHA2,* and *AtAHA3*, in *Arabidopsis* [8]. The transcript levels of PM AHA were found to be higher in a salt-tolerant poplar than a salt-sensitive poplar [9]. In addition, PM AHA mRNA is more abundant in halophytes than glycophytes [10, 11]. Salinity causes upregulation of PM *AHA* gene expression, as well as accelerates protein biosynthesis and H^+^-pumping activity in some plants [12–14]. AHA in a salt-tolerant rice species has higher activity than in a salt-sensitive rice species [15]. An *Arabidopsis* PM AHA4 mutant has dramatically reduced growth when exposed to salt stress compared to WT [16]. Expression of a constitutively activated PM AHA lacking the autoinhibitory domain in transgenic tobacco plants increases salt tolerance compared to untransformed plants [17].

*SOS1* genes have been found in many plants [18–25]. Of these, *Arabidopsis* SOS1 (AtSOS1) was the first PM Na^+^/H^+^ antiporter to be thoroughly physiologically, biochemically, and molecularly characterized [18, 26]. Exposure to salinity stress increases *SOS1* transcript abundance in wheat plants [19], induces the accumulation of *SOS1* mRNA in rice plants [27], and causes upregulation of *SOS1* transcription in *Arabidopsis* [28]. Under high salt conditions, *SOS1* mRNA levels are higher in *Thellungiella salsuginea* (a halophytic *Arabidopsis*-relative plant) than *Arabidopsis* [20]. Mutant *Arabidopsis* plants lacking SOS1 are extremely sensitive to salt stress [18, 29]. *Thellungiella salsuginea* lines expressing SOS1-RNAi (RNA interference) are sensitive to salt [20]. The salt sensitivity of an *Arabidopsis sos1* mutant can be overcome by transforming in native or other plant *SOS1* genes [27, 28]. *Arabidopsis* overexpressing *AtSOS1* is more salt tolerant than WT plants [30]. Expression of wheat SOS1 (*TaSOS1*) in transgenic tobacco plants improves their growth following NaCl treatment [31]. SOS1 uses the proton gradient established by PM AHA to exchange Na^+^ for H^+^ across the PM [3, 27]. The aforementioned data indicate the PM Na^+^/H^+^ antiporter SOS1 and H^+^-ATPase AHA are involved in plant salt tolerance, where an Na^+^/H^+^ antiporter utilizes the proton gradient generated by H^+^-ATPase to move Na^+^ from the cytoplasm to the external medium and help plant cells maintain non-toxic cytosolic concentrations of Na^+^. Therefore, theoretically, coordinating SOS1 and AHA could enhance Na^+^ extrusion, where co-expression of these two genes should confer better tolerance to salinity to transgenic plants. However, it has not been reported whether SOS1 and AHA1 function cooperatively in transgenic plants to more efficiently improve salinity tolerance.

*Sesuvium portulacastrum* is a halophyte that grows optimally in the presence of 200–300 mM NaCl [32]. When growing in a saline environment, *S. portulacastrum* cells accumulate large amounts of Na^+^ despite salt glands and bladders not being present in all tissues [33–35], suggesting *S. portulacastrum* may have a unique ability to remove Na^+^ from cells. The SOS1 protein functions as a PM Na^+^/H^+^ antiporter driven by the proton gradient that is produced by the PM H^+^-ATPase AHA, so they are considered as superior salt tolerance determinants [3, 36]. The *SpAHA1* and *SpSOS1* genes encode a PM H^+^-ATPase and Na^+^/H^+^ antiporter, respectively, and are more highly transcribed in *S. portulacastrum* plants exposed to salt stress. SpSOS1 more efficiently mediates Na^+^ removal using a proton gradient created by SpAHA1 in *SpAHA1*-*SpSOS1* co-transgenic yeast cells, where yeast cells co-expressing *SpSOS1* and *SpAHA1* grow better following NaCl treatment than cells transformed with only *SpSOS1* or *SpAHA1* [3]. Over-expression of *SpAHA1* conferred salt tolerance to transgenic *Arabidopsis* [37]. SpSOS1 complemented the salt sensitivity of transgenic *Arabidopsis sos1* mutant plants [38]. These results suggest that SpSOS1 and SpAHA1 are involved in salt tolerance of *S. portulacastrum*, and co-expression of *SpAHA1* and *SpSOS1* may improve transgenic plant salt tolerance. To test this hypothesis, *SpAHA1* and *SpSOS1* genes were co-transformed into *Arabidopsis* plants. Functional analyses indicate that *Arabidopsis* plants co-expressing *SpSOS1* and *SpAHA1* had better salt tolerance than plants expressing either gene alone due to efficient Na^+^ removal mediated by SpSOS1 using the extra proton gradient generated by SpAHA1. Therefore, genetic evidence may significantly guide development of more salt tolerant crops using PM-localized Na^+^/H^+^ antiporters and H^+^-ATPases.

## Results

### Transgenic plant identification

*SpSOS1* and *SpAHA1* were transformed alone or together into *Arabidopsis* plants using *Agrobacteria* carrying pCAMBIA1304-*SpSOS1*, pCAMBIA1304-*SpAHA1,* or pCAMBIA1304-*SpSOS1*-*SpAHA1*. PCR analyses of genomic DNA performed using *SpAHA1/SpSOS1* and *hygB* gene-specific primers revealed 12 *SpSOS1*-, 11 *SpAHA1*-, and 10 *SpSOS1*-*SpAHA1*-transgenic lines were obtained (Additional file 1: Figure S1**).** Total RNA was isolated from the above transgenic plant lines and RT-PCR analyses were used to study the *SpAHA1* and *SpSOS1* expression levels. The *SpAHA1* gene was significantly expressed in all single *SpAHA1*-transgenic lines, except for SpAHA1- lines 5 and 8. Of the *SpSOS1*-expressing single transgenic plants, *SpSOS1*-line 1 had the highest *SpSOS1* expression of the *SpSOS1*-transgenic lines. In *SpAHA-SpSOS1* co-expressing plants, the clearest expression of both *SpAHA1* and *SpSOS1* was observed in line 10 (Additional file 2: Figure S2). Therefore, the T3 generation transgenic plants of the homozygous *SpSOS1*-line 1, *SpAHA1*-line 1, and *SpAHA1-SpSOS1*-line 10 were used to characterize the functions of SpSOS1 and SpAHA1.

### SpSOS1 and SpAHA1 functioned together to more efficiently improve transgenic plant salt tolerance

**Fig. 1:**
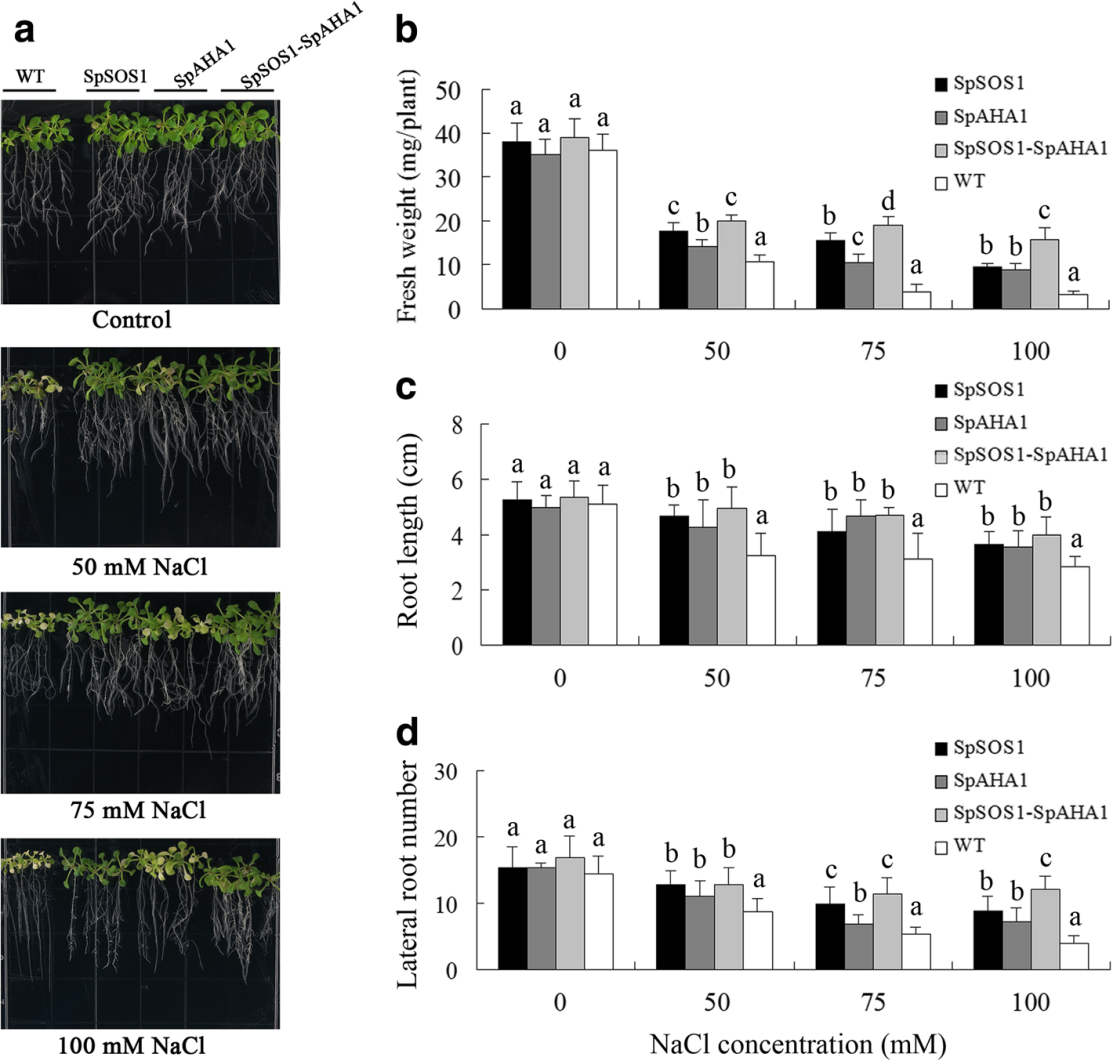
Growth of transgenic and WT seedlings under salt stress. Five-day-old seedlings grown on MS plates were transferred to MS plates containing 0, 50, 75, and 100 mM NaCl. a The seedlings were photographed after 2 weeks of growth. The growth was assessed based on fresh weight (b), root length (c), and number of lateral roots (d). Data are presented as mean ± SE of 12 replicates, where the different letters above the columns indicate statistically significant differences at a *p* < 0.05 level between the experimental cohorts. SpSOS1, *SpSOS1*-overexpressing plants; SpAHA1, *SpAHA1*-overexpressing plants; SpSOS1-SpAHA1, *SpSOS1* and *SpAHA1* co-expressing plants; WT, wild-type plants

In plant cells, the PM Na^+^/H^+^ antiporter SOS1 mediates Na^+^ excretion using a proton gradient created by PM H^+^- ATPases. Therefore, both of these proteins are involved in plant salt tolerance. Much evidence indicates that overexpressing *SOS1* or *AHA* increases the salt tolerance of transgenic plants [39]. In addition, our recent investigation found SpSOS1 and SpAHA1 function cooperatively in transgenic yeast cells, where yeast cells co-expressing *SpSOS1* and *SpAHA1* are better growers than cells transformed with only *SpAHA1* or *SpSOS1* [3]. Therefore, we hypothesized co-expression of *SpSOS1* and *SpAHA1* would increase the salt tolerance of transgenic plants compared to plants transformed with only *SpSOS1* or *SpAHA1*. To examine the influence of *SpSOS1*-*SpAHA1* co-expression on the salt tolerance of transgenic plants, 5-day-old *Arabidopsis* WT, *SpSOS1-*expressing, *SpAHA1*-expressing, and *SpSOS1*-*SpAHA1* co-expressing seedlings were grown on MS plates containing 0, 50, 75, or 100 mM NaCl. Two weeks post-NaCl treatment, the seedlings were photographed and their fresh weight, root length, and lateral root number were measured**.** Upon exposure to salinity stress, the growth of all tested plants decreased, but expression of either *SpSOS1* or *SpAHA1* ameliorated this growth inhibition from NaCl treatment compared to WT plants. Furthermore, among all the transgenic plants, salt tolerance improved the most in plants co-expressing *SpSOS1* and *SpAHA1* based on growth in MS medium containing different concentrations of NaCl (Fig. 1).

**Fig. 2:**
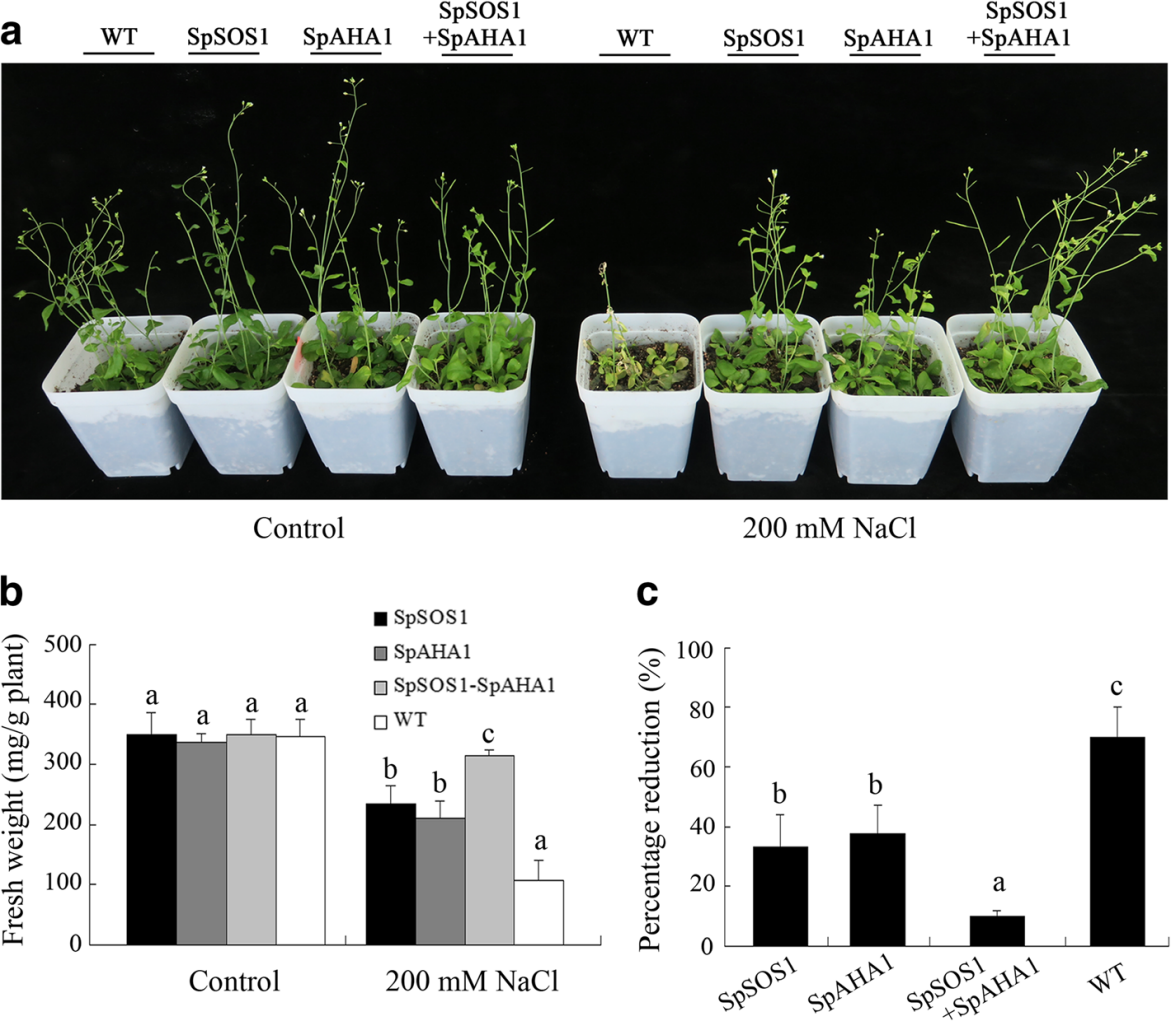
Growth of transgenic and WT seedlings. Seven-day-old WT and transgenic seedlings were transferred from MS plates into soil (4 plants/pot) grown for 4 weeks. The plants were then treated with 0 or 200 mM NaCl. Ten days post-treatment, the plants were photographed (a) and their fresh weight was measured (b). The two preparations were performed at different times to more accurately assess the salt tolerance of the transgenic plants and (c) the relative change (percentage reduction) in fresh weight in the presence of salt stress relative to the nonstressed control was determined. Data are presented as mean ± SE of nine replicates. Different letters above the columns indicate statistically significant differences at a *p* < 0.05 level among the different experimental cohorts. SpSOS1, *SpSOS1*-overexpressing plants; SpAHA1, *SpAHA1*-overexpressing plants; SpSOS1 + SpAHA1, *SpSOS1* and *SpAHA1* co-expressing plants; WT, wild-type plants

Similarly, the growth of transgenic and WT plants was inhibited in soil supplemented with 200 mM NaCl. However, *Arabidopsis* plants expressing both *SpSOS1* and *SpAHA1* grew the best among the different experimental cohorts under these conditions (Fig. 2a). *SpAHA1-SpSOS1*-line 10 displayed 26, 33, and 67% greater fresh weights than *SpSOS1*-line 1, *SpAHA1*-line 1, and WT plants, respectively (Fig. 2b). The percent reduction in growth of plant lines treated with NaCl was ordered: *SpSOS1*-*SpAHA1* co-expressing plants <*SpSOS1*-expressing plants<*SpAHA1*-expressing plants <WT plants (Fig. 2c). These findings indicate the PM-localized Na^+^/H^+^ antiporter SpSOS1 and H^+^-ATPase SpAHA1 function cooperatively to improve the salt tolerance of transgenic plants.

### *SpSOS1*-*SpAHA1* co-expressing *Arabidopsis* plants had higher H^+^ efflux rates than *SpSOS1-* or *SpAHA1-*expressing plants under high saline conditions

**Fig. 3:**
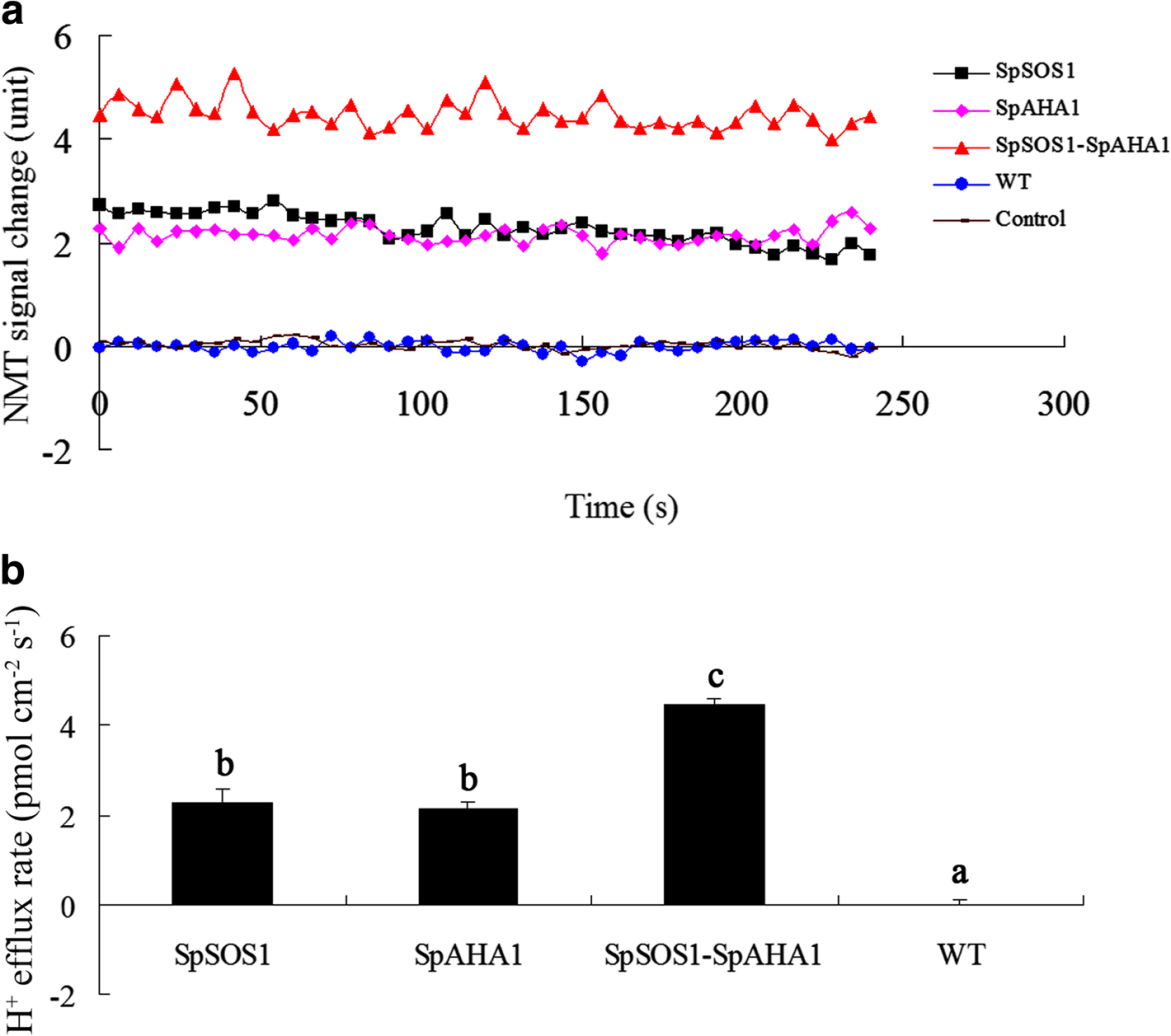
H^+^ flux in roots of NaCl-treated *Arabidopsis* plants. Seedlings were grown for 3 days on MS plates supplemented with 100 mM NaCl and then H^+^ flux in the roots was measured using the non-invasive micro-test technology (NMT) technique described in the Methods section. (a) Changes in the NMT signals are expressed as arbitrary units. (b) H^+^ flux is expressed as the amount of efflux per second per square centimeter (pmol•cm^− 2^•s^− 1^). Data are presented as mean ± SE of six replicates. Different letters above the columns indicate statistically significant differences at a *p* < 0.05 level among the different experimental cohorts. SpSOS1, *SpSOS1*-overexpressing plants; SpAHA1, *SpAHA1*-overexpressing plants; SpSOS1-SpAHA1, *SpSOS1* and *SpAHA1* co-expressing plants; WT, wild-type plants; Control, mock controls

Net H^+^ flux at the roots of WT plants was close to the mock control (Fig. 3A), which is in full agreement with the recent report that both transient and long-term salinity exposure did not induce H^+^ efflux from *Arabidopsis* roots [8]. These results suggested that H^+^ efflux might be balanced by H^+^ influx at the roots exposed to salinity stress. PM H^+^-ATPase activity is a major factor in H^+^ excretion at the PM [40]. It was recently reported that SpAHA1 can function as an H^+^-ATPase on vesicles isolated from yeast cells expressing *SpAHA1* [3]. Roots expressing SpAHA1 had a faster net H^+^ efflux than the WT plants under saline conditions, suggesting SpAHA1 is responsible for the extra H^+^ efflux, i.e., SpAHA1 pumped more protons out of the cells. It is not expected that protons were extruded faster in roots transformed with *SpSOS1* relative to WT plants and the phenomenon might be from feedback regulation of Na^+^ extrusion mediated by SpSOS1. This hypothesis is also supported by the H^+^ flux in the roots of *SpSOS1*-*SpAHA1* co-expressing transgenic plants (Fig. 3) being the highest among all the transgenic plants, where the H^+^ efflux rates in the roots co-expressing *SpSOS1*-*SpAHA1* were 49 and 52% greater than *SpSOS1*- and *SpAHA1*-expressing roots, respectively.

### Plants co-expressing *SpSOS1-SpAHA1* had higher Na^+^ efflux in roots and less Na^+^ accumulation after NaCl treatment

**Fig. 4:**
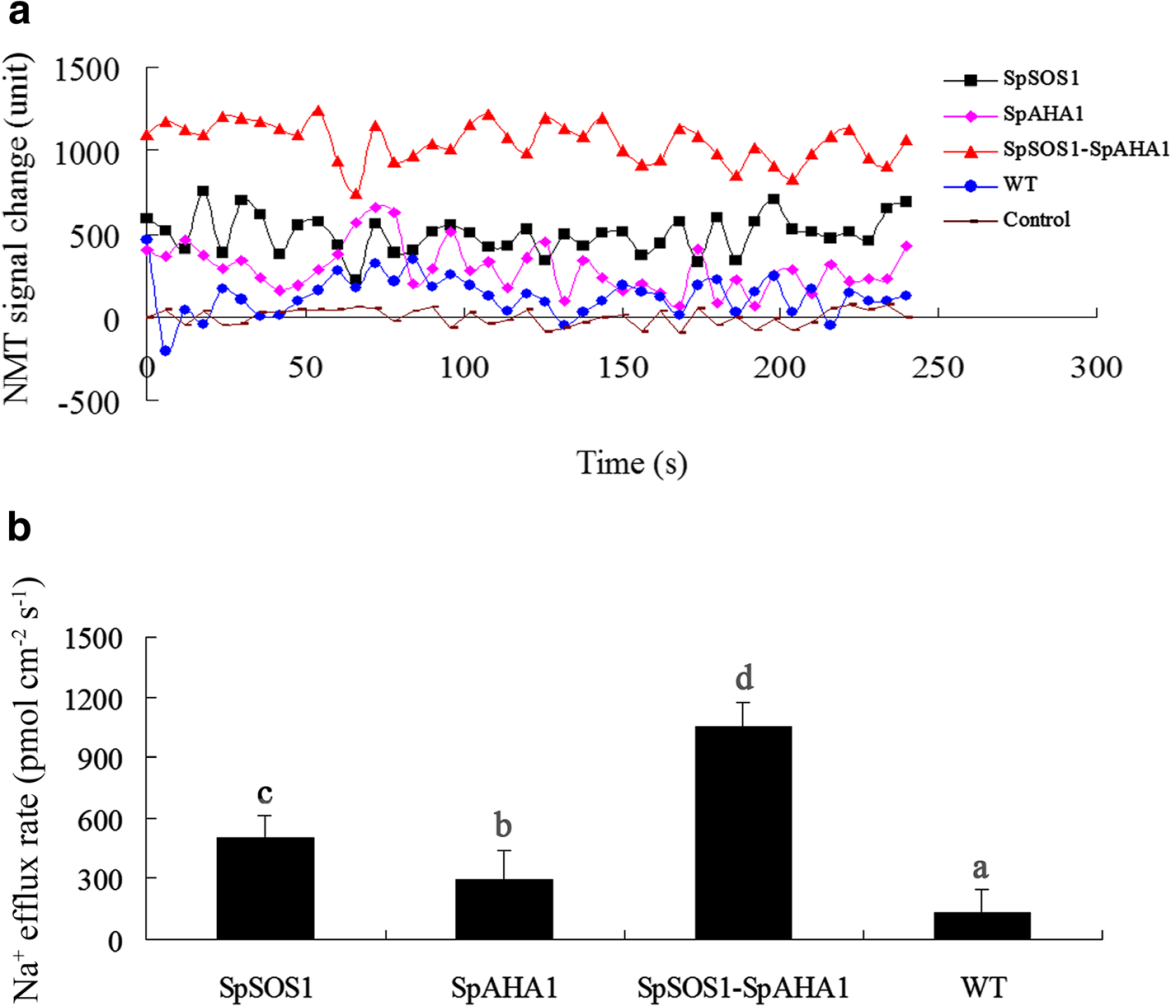
Na^+^ flux in roots of NaCl-treated *Arabidopsis* plants. Seedlings were grown for 3 days on MS plates containing 100 mM NaCl. Na^+^ flux in the roots was then measured using the NMT technique described in the Methods section. (a) Changes in the NMT signals are expressed as arbitrary units. (b) Na^+^ flux is expressed as the amount of efflux per second per square centimeter (pmol•cm^− 2^•s^− 1^). Data are presented as mean ± SE of six replicates. Different letters above the columns indicate statistically significant differences at a *p* < 0.05 level among the different experimental cohorts. SpSOS1, *SpSOS1*-overexpressing plants; SpAHA1, *SpAHA1*-overexpressing plants; SpSOS1-SpAHA1, *SpSOS1* and *SpAHA1* co-expressing plants; WT, wild-type plants; Control, mock controls

**Fig. 5:**
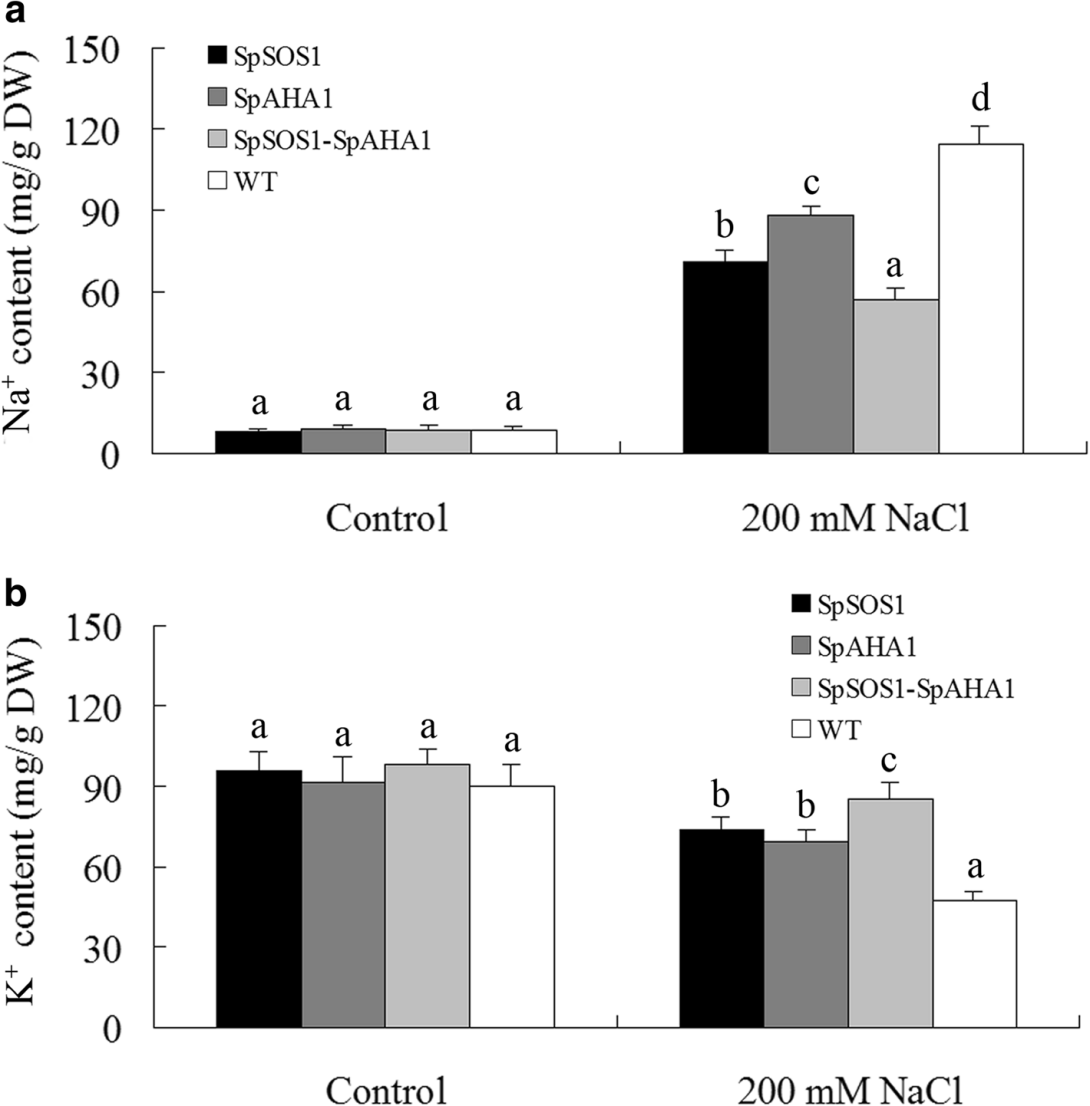
Na^+^ and K^+^ content in *Arabidopsis* plants. WT and transgenic plants were grown for 10 days in soil containing 0 or 200 mM NaCl. Na^+^ (a) and K^+^ (b) in the leaves of these plants were measured as described in the Methods section. Data are presented as mean ± SE of three replicates. Different letters above the columns indicate statistically significant differences at a *p* < 0.05 level among the different experimental cohorts. SpSOS1, *SpSOS1*-overexpressing plants; SpAHA1, *SpAHA1*-overexpressing plants; SpSOS1-SpAHA1, *SpSOS1* and *SpAHA1* co-expressing plants; WT, wild-type plants

SOS1 mediates Na^+^ excretion from cells and is a key halotolerance factor. SpSOS1 has been shown to be a PM-localized Na^+^/H^+^ antiporter and capable of improving the growth of transgenic yeast cells under salt stress by decreasing the cellular Na^+^ content [3]. In this scenario, the roots from all tested plants grown in medium without NaCl displayed Na^+^ uptake characteristics, but no significant differences in Na^+^ flux activities at roots of transgenic and untransformed plants under unstressed condition were observed (Additional file 3: Figure S3). On the contrary, NaCl treatment stimulated Na^+^ effluxes at all tested roots. *SpSOS1*-expressing roots had faster Na^+^ efflux relative to WT plants in saline conditions (Fig. 4a, b), suggesting the extra Na^+^ extrusion may be mediated by SpSOS1, which would result in the observed lower Na^+^ content in the *SpSOS1*-transgenic plants than WT plants under salt stress (Fig. 5a). SOS1-mediated Na^+^/H^+^ exchange is powered by a proton gradient generated by an H^+^-ATPase. Therefore, a proton gradient generated by SpAHA1 (Fig. 3) might catalyze native SOS1 (AtSOS1) to transport more Na^+^ out of cells, which may be one reason for the higher Na^+^ efflux rate in roots transformed with *SpAHA1* compared to WT plants in saline conditions (Fig. 4). Roots co-expressing *SpAHA1* and *SpSOS1* had the highest Na^+^ efflux rates among all the transgenic plant lines tested, where the Na^+^ efflux rate in *SpSOS1*-*SpAHA1* co-transgenic roots was 53 and 72% greater than the plants expressing *SpSOS1* or *SpAHA1* singly, respectively (Fig. 4b). Correspondingly, the Na^+^ levels in the transgenic plants were lower than in WT plants (Fig. 5a). Therefore, it is reasonable that *SpSOS1*-*SpAHA1* co-expression quickened Na^+^ extrusion in the roots of and decreased Na^+^ accumulation in transgenic plants compared to *SpSOS1-* or *SpAHA1-*expressing plants under saline conditions (Fig. 4, 5a). These results indicate SpAHA1 produced an additional proton gradient and, thus, promoted SpSOS1-mediated Na^+^ extrusion in *Arabidopsis* plants co-expressing both the *SpAHA1* and *SpSOS1* genes.

### *SpSOS1-SpAHA1* co-transgenic plants had higher K^+^ retention under saline conditions

Among the physiological and biochemical processes in plant cells influenced by high salinity, nutrient imbalance is among the most deleterious resulting effects [41]. The chemical and physical characteristics of sodium most resemble potassium among the nutrient elements. Therefore, excess Na^+^ inhibits plant growth by interfering with cytosolic K^+^ homeostasis. No differences in K^+^ content was found among all the tested plants under normal conditions. However, upon salinity stress, the transgenic plants displayed less of a decrease in K^+^ content than the WT plants. Furthermore, co-expression of *SpSOS1* and *SpAHA1* most efficiently alleviated K^+^ loss among the transgenic plants exposed to NaCl, where the K^+^ content was highest in the leaves of plants co-expressing *SpSOS1* and *SpAHA1* (85 mg/g dry weight), followed by those from *SpSOS1*-transgenic plants (73 mg/g dry weight), and then *SpAHA1*-expressing plants (69 mg/g dry weight). WT plants had the lowest K^+^ content (47 mg/g dry weight; Fig. 5b).

### *SpSOS1-SpAHA1* co-expression decreased malondialdehyde accumulation in transgenic plants

**Fig. 6:**
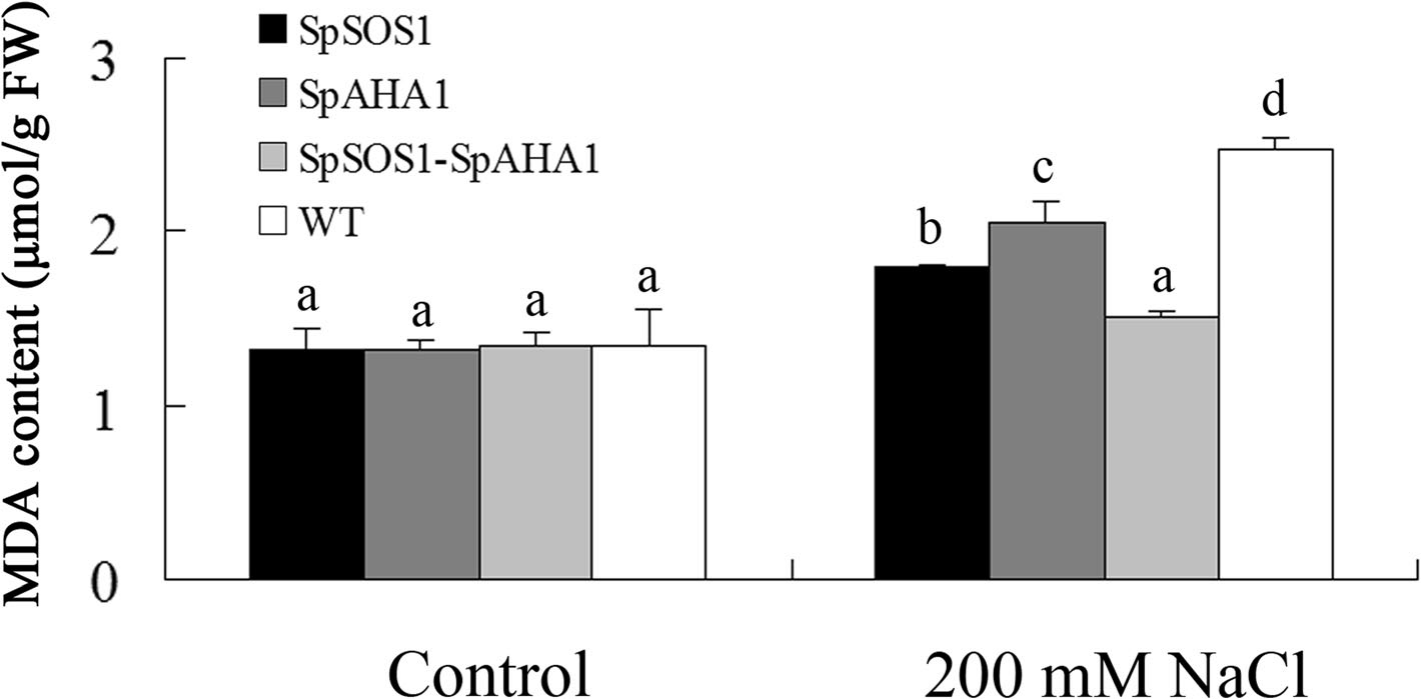
Malondialdehyde content in leaves. Two-week-old WT and transgenic *Arabidopsis* seedlings were treated with 200 mM NaCl for 7 days and then their leaves were harvested. Malondialdehyde content in the leaves was measured as described in the Methods section. Data are presented as mean ± SE of three replicates. Different letters above the columns indicate statistically significant differences at a *p* < 0.05 level among the different experimental cohorts. SpSOS1, *SpSOS1*-overexpressing plants; SpAHA1, *SpAHA1*-overexpressing plants; SpSOS1-SpAHA1, *SpSOS1* and *SpAHA1* co-expressing plants; WT, wild-type plants

Salinity creates oxidative stress and excess reactive oxygen species can interfere with metabolism in the cytoplasm, such as by damaging membrane structures and destroying membrane integrity through lipid peroxidation [42]. An indicator of membrane lipid oxidation, malondialdehyde (MDA) represents membrane lipid damage to some extent. Upon exposure to NaCl, the amount of MDA in the leaves of all tested plants increased, but MDA accumulation in the *SpSOS1*-*SpAHA1* co-expressing leaves was the lowest (Fig. 6) at only 84, 74, and 61% of that in *SpSOS1*-expressing, *SpAHA1*-expressing, and WT plants under saline conditions. These results indicate *SpSOS1* and *SpAHA1* coordination could more efficiently reduce oxidative damage to membranes from salinity stress in transgenic plants.

## Discussion

Plants grown under K^+^ deficiency substitute it by Na^+^, especially some halophyte species can use Na^+^ for stomata operation instead of K^+^ [43]. However, when there is excess sodium in the cytosol, it interferes with some key metabolic processes and eventually inhibits the growth and development of the plant. To tolerate high Na^+^ levels, plant cells must be capable of removing Na^+^ from the cytoplasm through some physiological processes. In one of these processes, cytoplasmic Na^+^ can be imported into vacuoles through tonoplast Na^+^/H^+^ antiporter NHXs using an electrochemical gradient established by a vacuolar H^+^-ATPase and H^+^-PPase (AVP/VP) using protons. The sequestration of Na^+^ into vacuoles not only prevents the deleterious effects resulting from Na^+^ in the cytoplasm, but also lets the plants use Na^+^ as an osmoticum, which helps maintain the osmotic potential that drives water into the cells [39, 44]. Therefore, tonoplast NHX antiporters and H^+^-pumps have important functions in plant responses to salt stress. In transgenic plants, overexpression of genes encoding vacuolar Na^+^/H^+^ antiporters or H^+^-PPases enhances salt tolerance [39, 42]. Furthermore, co-expression of AVP and NHX may better improve the growth of transgenic plants exposed to salt stress through more efficient compartmentalization of Na^+^ into vacuoles than when NHX or AVP are expressed alone. Co-expression of *ZxNHX* and *ZxVP1–1* confers better salt tolerance to transformed sugar beet and lotus plants [42, 45]. *NHX1-AVP1* co-transgenic rice grows better under salt stress than rice plants expressing only one of these genes [46, 47]. Tobacco plants co-expressing *TNHXS1* and *TVP1* have higher salt tolerance than transgenic plants expressing *TNHXS1* or *TVP1* alone [48]. Another mechanism contributing to Na^+^ extrusion is the PM Na^+^/H^+^ antiporter SOS1. The Na^+^ extrusion mediated by SOS1 is also driven by electrochemical gradients of protons generated by a PM H^+^-pump (H^+^-ATPase, AHA). The overexpression of *SOS1* significantly improves the salt tolerance of transgenic grapevine compared to WT plants [49]. Overexpression of the *SOS1* gene in tobacco plants increases salt tolerance by maintaining a lower Na^+^ content [50] and the growth of *Arabidopsis* plants overexpressing *SOS1* is better than that of WT plants under salt stress [51]. It has been reported that overexpression of *PeHA1*(H^+^-ATPase 1)*,* a poplar gene encoding a PM-localized H^+^-ATPase, enhances the salt tolerance of transgenic *Arabidopsis* [52]. These studies suggest that co-expression of both *SOS1* and *AHA* in transgenic plants should more effectively increase salinity tolerance just as co-expression of vacuolar NHX and AVP results in higher salt tolerance. In the present investigation, *SpAHA1*-transgenic roots had faster H^+^ efflux than WT plants under salt stress (Fig. 3), suggesting SpAHA1 enhanced proton efflux and generated an additional proton gradient that acted as a driving force for Na^+^/H^+^ exchange mediated by SpSOS1. More interestingly, the magnitude of net H^+^ flux is in 3 to 4 pmol•cm^− 2^•s^− 1^ range (Fig. 3), while Na^+^ flux is around 1000 pmol•cm^− 2^•s^− 1^; the stoichiometric ratio for Na^+^/H^+^ exchange of SOS1 protein is 1H^+^:1Na^+^, so such tiny increase in H^+^ flux (from 2 to 4 pmol•cm^− 2^•s^− 1^) may not cause such a massive flux of Na^+^. Net H^+^ fluxes at roots was determined in the present study, that is, the data for net H^+^ efflux is equal to total H^+^ efflux minus total H^+^ influx at the roots, suggesting that H^+^-ATPase mediated H^+^ efflux is likely balanced by H^+^ uptake through SOS1 transporters. These suggest that SpAHA1 might provided more H^+^ gradient than the shown data of net H^+^ efflux in transgenic plant exposed to salinity stress, resulting in faster Na^+^ efflux from cells, consequently Na^+^ content in transgenic plants was lower than that in wild type plants (Fig. 5a). Overexpression of *SpSOS1* in transgenic *Arabidopsis* accelerated Na^+^ efflux in the roots (Fig. 4a, b), resulting in decreased Na^+^ content in the transgenic plants compared to the WT plants (Fig. 5a). Interestingly, the rate of H^+^ efflux in the *SpSOS1-*expressing roots was faster than in the WT or *SpAHA1*-transgenic roots (Fig. 3), suggesting that increased Na^+^ extrusion mediated by SpSOS1 might regulate H^+^-pumping activity via feedback at the PM. This is because the Na^+^/H^+^ exchange mediated by SOS1 is dependent on energy and driven using a PM-localized H^+^-ATPase-driven proton motive force [53]. The H^+^ and Na^+^ efflux rates in the roots of *Arabidopsis* plants co-expressing *SpAHA1* and *SpSOS1* were highest among all the transgenic plants (Figs. 3 and 4), leading to the lowest Na^+^ content in the co-transformed plants relative to other transgenic plants expressing only *SpSOS1* or *SpAHA1* (Fig. 5a). In response to NaCl treatment, the biomass of the transgenic *Arabidopsis* plants co-expressing *SpSOS1* and *SpAHA1* was greater than the biomasses of the single-gene expressing plants (Fig. 2). Taken together, the higher rate of Na^+^ extrusion, lower Na^+^ levels, and better growth of the *SpAHA1*-*SpSOS1* co-expressing plants compared to the single *SpAHA1* or *SpSOS1* gene transgenic plants provides direct genetic evidence that SOS1 and AHA function in a cooperative manner to inhibit Na^+^ accumulation in the cytosol and play important roles in plant adaption to highly saline conditions.

High soil salinity is characterized by high soluble salt concentrations, of which sodium salt is the most soluble and widespread salt [44]. Excessive sodium ions in soils can enter into plant cells and then interference with some critical biochemical and physiological processes. The most deleterious effect of salinity is ion toxicity [41]. K^+^ is a necessary macronutrient that has a critical role in the growth and development of plants [54]. Due physicochemical similarities between Na^+^ and K^+^, Na^+^ can compete with K^+^ for binding sites important in critical cytoplasmic physiological and biochemical processes [55]. In particular, Na^+^ inhibits the activity of many K^+^-dependent enzymes [56] and, therefore, excess Na^+^ can inhibit K^+^-associated activities in the cytosol [55]. It is hypothesized that plant survival in the presence of salt stress requires a high K^+^/Na^+^ ratio in the cytoplasm [57]. Therefore, limiting Na^+^ influx into cells may facilitate plant growth under salt stress [58, 59]. Under high salt conditions, the PM potential becomes depolarized, which encourages passive Na^+^ influx into cells and K^+^ efflux out of cells. H^+^-ATPase-generated electrochemical potential gradients across PMs can repolarize PMs following NaCl-induced depolarization [39]. Therefore, maintenance of the PM potential using H^+^-ATPases can reduce the Na^+^ influx via depolarization-activated non-selective cation channels (NSCCs) and K^+^ efflux via K^+^ outward rectifiers (KORs) and NSCCs [60]. The net H^+^ efflux from root cells in *SpAHA1*-transgenic plants occurred at a higher rate relative to WT plant roots, suggesting SpAHA1 increased the H^+^-ATPase activity and electrochemical potential and, thus, the H^+^ gradient across the PM. This may reduce Na^+^ influx and K^+^ efflux and, correspondingly, the *SpAHA1*-transgenic plants had less Na^+^ and more K^+^ than WT plants (Fig. 5a, b). In addition to Na^+^ extrusion, SOS1 is also involved in K^+^ homeostasis in plant cells under high salt conditions. Transgenic tobacco plants expressing *SbSOS1* contain less Na^+^, but more K^+^, in their roots than WT plants under high salt stress [50]. Horie et al. [61] suggested SOS1 plays a primary role facilitating high-affinity absorption of K^+^ into roots. SOS1 is necessary for protecting K^+^ uptake and is involved in K^+^ homeostasis maintenance in cells under salinity stress [19, 62]. Overexpression of *TaSOS1* confers salt tolerance to transgenic tobacco plants by decreasing the Na^+^ and increasing the K^+^ levels [31]. *Arabidopsis* roots expressing *SpSOS1* displayed faster Na^+^ efflux than WT plant roots under saline condition (Fig. 4), suggesting SpSOS1 was responsible for the extra Na^+^ extrusion. The faster H^+^ efflux in the roots of plants expressing *SpSOS1* may aid repolarization following NaCl-induced depolarization of the PM, thus decreasing Na^+^ influx and K^+^ efflux [60]. These actions may have led *SpSOS1*-transgenic plants to contain less Na^+^ and more K^+^ relative to WT plants under salt treatment. Therefore, faster H^+^ and Na^+^ efflux in the roots also resulted in retention of more K^+^ and a lower Na^+^ concentration in cytosol of *SpSOS1*-*SpAHA1* co-transgenic *Arabidopsis* plants compared to plants expressing *SpSOS1* or *SpAHA1* alone (Figs. 3 and 4). This led to *Arabidopsis* plants co-expressing *SpSOS1* and *SpAHA1* to have higher a K^+^/Na^+^ level than the transgenic plants with only *SpSOS1* or *SpAHA1*, which is strong evidence of salt tolerance. These results suggest SOS1 and AHA1 facilitate more efficient prevention of K^+^ loss and enhance Na^+^ extrusion and thereby contribute to better salt tolerance.

Another deleterious effect of salinity stress in plants is associated with oxidative stress [39]. Accumulation of ROS (reactive oxygen species) is toxic in cells. Therefore, intracellular ROS levels are tightly regulated under normal conditions through a number of intracellular peroxidative and antioxidative reactions within the cell. Salinity can disrupt the ROS production and scavenging balance, resulting in ROS accumulation, which can negatively affect cellular structures and metabolism [63, 64]. In order to protect cells from salinity-induced oxidative damage, excess ROS is scavenged by antioxidant molecules and enzymes. RCD1 (Radical-induced cell death) is a regulator of responses to oxidative stress and protects cells from oxidative damage caused by H_2_O_2_, diamide, and tert-butyl peroxide [65–67]. SOS1 functions in tolerance to oxidative stress by interacting with RCD1 and regulating expression of certain genes associated with oxidative-stress tolerance in *Arabidopsis* [66]. Haem oxygenase (HO) is an important factor in plant antioxidant defense systems. Overexpression of the *AtHO* gene enhances *Arabidopsis* tolerance to salt by increasing PM H^+^-ATPase activity and expression [68]. Excess ROS can damage membrane structures by oxidizing lipids in the PM, leading some key metabolites abnormally leak out of cells. ROS could disturb ion homeostasis in cells by inducing the efflux of several cations [69–71]. Coskun et al. [53] found NaCl-induced efflux of K^+^ was a result of a lack of PM integrity in rice. This indicates the maintenance of PM stability has a key role in plant salt tolerance. In the present investigation, both SpSOS1 and SpAHA1 prevented the accumulation of MDA in transgenic plants following NaCl treatment, but plants co-expressing *SpSOS1* and *SpAHA1* had a more drastic decrease in MDA content under salt stress than plants expressing only one of these genes. This suggests SpSOS1 and SpAHA1 coordinate in transgenic *Arabidopsis* and ameliorate salt toxicity by more efficiently alleviating oxidative damage to the PM generated by salinity stress.

## Conclusions

*Arabidopsis* plants co-expressing *SpSOS1* and *SpAHA1* had higher K^+^ and lower MDA levels than plants transformed with only *SpSOS1* or *SpAHA1* and, thus, grew better under salt stress. The coordinated action of these genes might be a novel and effective method for increasing the salt tolerance of crops.

## Methods

### Plasmid construction

The *SpAHA1* and *SpSOS1* genes were separately cloned from *S. portulacastrum* and inserted into plasmids p414 (p414-*SpAHA1*) and p416 (p416-*SpSOS1*) in our recent investigation [3]. Plant vectors expressing *SpAHA1* or *SpSOS1* alone or together bicistronicly were constructed as described in Additional file 4: Figure S4. (1) Amplification of the *SpSOS1* gene was performed using the p416-*SpSOS1* plasmid as the template and the primers SpSOS1-F and SpSOS1-R (Additional file 5: Table S1). The amplified gene was inserted into the pCAMBIA1300 vector between *Sal* I and *Kpn* I restriction sites, generating pCAMBIA1300-*SpSOS1*. (2) A fragment containing a constitutive promoter (cauliflower mosaic virus 35S promoter), the *SpSOS1* gene, and the NOS terminator was excised from the pCAMBIA1300-*SpSOS1* plasmid using *Pst* I and *Eco*R I restriction enzymes and transferred into the plant expression vector pCAMBIA1304 between the same restriction sites. The resulting plasmid was named pCAMBIA1304-*SpSOS1*. (3) The *SpAHA1* gene was amplified using the p414-*SpAHA1* plasmid as the template and the primers SpAHA1-F and SpAHA1-R (Additional file 5: Table S1) and inserted into the pCAMBIA1304 and pCAMBIA1304-*SpSOS1* plasmids between the *Spe* I and *Eco*72 I restriction sites to replace the *GUS* (β-glucuronidase) gene. The resulting plasmids were designated pCAMBIA1304-*SpAHA1* and pCAMBIA1304-*SpSOS1*-*SpAHA1*, respectively. The plasmids were all verified by sequencing.

### *Arabidopsis* transformation and identification

The three recombinant plasmids described above were added to a 100 mM CaCl_2_ solution containing competent *Agrobacterium tumefaciens* GV3101 cells. The plasmids were then introduced into the *Agrobacterium* cells via heat shock (42 °C). Finally, the above three expression cassettes were transformed into *Arabidopsis thaliana* (Col-0) by infecting flower buds with the *Agrobacterium* cells containing the recombinant plasmids [72]. T0-generation seeds were screened initially on MS (Murashige & Skoog) medium supplemented with 50 μg/mL hygromycin B. DNA was purified from the candidate lines and used as template for PCR (polymerase chain reaction) amplification with specific primer pairs to identify the different transgenic plants. The primers are listed in Additional file 5: Table S1. All the transgenic lines were furthermore verified by PCR using primers specific for the *hygB* marker gene, HygB-TF and HygB-TR (Additional file 5: Table S1). Total RNA was purified from the transgenic lines and *SpAHA1* and *SpSOS1* expression was assessed by RT-PCR (reverse transcription PCR) with a housekeeping gene, *Actin,* as an internal control. The primers for the *SpAHA1* (SpAHA1-RT-F and SpAHA1-RT-R), *SpSOS1* (SpSOS1-RT-F and SpSOS1-RT-R), and *Actin* (Actin-RT-F and Actin-RT-F) genes are listed in Additional file 5: Table S1**.** The PCR conditions were as follows: 94 °C for 2 min, followed by 28 cycles of 94 °C for 30 s, 55 °C for 30 s, and 72 °C for 30 s, and a final extension at 72 °C for 5 min. The resulting PCR products were assessed by agarose gel electrophoresis.

### Cultivation and salt treatment of transgenic and WT plants

To analyze the salt tolerance of transgenic and WT plants, seeds from T3 homozygous transgenic lines (expressing single *SpSOS1*, single *SpAHA1,* and both *SpSOS1* and *SpAHA1*) and untransformed plants were germinated on MS plates in a growth chamber (22 °C with a 16 h light / 8 h dark cycle and a light intensity of 100 μmol•m^− 2^•s^− 1^). After 5 days, the seedlings grown on MS plates were transferred onto MS plates containing 0, 50, 75, and 100 mM NaCl and allowed to grow for 2 weeks. Then the root length, number of lateral roots, and fresh seedling weights were measured. In addition, 10-day-old seedlings were transferred to a mixture of organic soil and sand (3:1, *v*/v) in pots (4 seedlings/pot) and grown in a greenhouse with long-day conditions (16 h light/8 h dark at 22 °C and a light strength of 150 μmol•m^− 2^•s^− 1^) for 4 weeks. The pots containing the plants were then put into water containing 0 or 200 mM NaCl. Ten days post-NaCl treatment, the treated plants were photographed and their fresh weights were determined.

### Determination of Na^+^ and K^+^ content in *Arabidopsis* plants

At the end of the NaCl treatment, the WT and transgenic plants were separately collected. The Na^+^ and K^+^ in the samples were measured using atomic absorption spectrometry as described in a previous work [31].

### Measurement of Na^+^ and H^+^ flux in roots

Seven-day-old uniform T3 seedlings, which had been grown on MS plates, were transferred to MS medium containing 100 mM NaCl and grown for 3 days, and then the roots of salt stressed seedlings were put into measurement buffer to balance for 10 min, after that net H^+^ and Na^+^ fluxes were measured in the YoungerUSA Xuyue (Beijing) BioFunction Institute by using Non-invasive Micro-test Technology (NMT100 Series, Xuyue (Beijing) Sci. & Tech. Co., Ltd., Beijing, China) Software.

H^+^, Na^+^-selective microsensors were prepared as described previously [60]. Pre-pulled and silanized microsensor (Φ4.5 ± 0.5 μm, XY-CGQ-01, YoungerUSA) were first filled with a backfilling solution (H^+^: 15 mM NaCl + 40 mM KH_2_PO_4_, pH 7.0; Na^+^: 250 mM NaCl) to a length of approximately 1.0 cm from the tip. Then the microsensors were front filled with 40–50 μm columns of selective liquid ion-exchanger (LIX) (H^+^: XY-SJ-H; Na^+^: XY-SJ-Na; all from YoungerUSA). An Ag/AgCl wire microsensor holder YG003-Y11 (YoungerUSA) was inserted in the back of the microsensor to make electrical contact with the electrolyte solution. YG003-Y11 (YoungerUSA) was used as the reference microsensor. Prior to the flux measurement, the microsensor were calibrated with cultural media having different concentrations of H^+^ (pH 6.0 and pH 7.0) or Na^+^ (5 mM and 0.5 mM), respectively. Only microsensor with a Nernstian slope >50 mV/decade were used in our study. The same microsensors were calibrated again according to the same procedure and standards after each test. After that, net ion fluxes were recorded on the root meristematic zones, 120 μm from the tip where SOS1 activity was the highest [73], in 5 mL measurement buffer (0.1 mM KCl, 0.1 mM CaCl_2_, 0.1 mM MgCl_2_, 0.5 mM NaCl, 0.3 mM MES (2-(N-Morpholino) ethanesulfonic acid) and 0.2 mM Na_2_SO_4_, pH 6.5). Net H^+^ and Na^+^ flux was calculated by Fick’s law of diffusion [60]. Six biological repeats were performed for each analysis.

### Assays of malondialdehyde content

Two-week-old T3 transgenic and untransformed *Arabidopsis* seedlings were grown in the presence of 200 mM NaCl for 7 days. Malondialdehyde (MDA) content in the leaves was measured using the thiobarbituric acid method previously described by Dhindsa and Matowe [74].

### Statistical analysis

Two-tailed Student’s t-tests were used to analyze the data. The results are expressed as mean ± SE and differences with a *P*-value < 0.05 were considered statistically significant. At least three biological replicates were performed for each experiment.

## Additional files

Additional file 1: **Figure S1.** Molecular identification of transgenic plants. DNA was purified from transgenic and WT plant leaves. (a) PCR identification of *SpSOS1* transgenic plants. M: DL2000 marker (Sangon Biotech, China; No. B600335); 1–12, different transgenic lines (lines 1–12); 13, negative control (WT plants). (b) PCR identification of *SpAHA1* transgenic plants. M: DL2000 marker; 1–11, different transgenic lines (lines 1–11); 12, negative control (WT plants). (c) PCR identification of *SpSOS1* and *SpAHA1* co-expressing plants. M: DL2000 marker; 1–10, different transgenic lines (lines 1–10); 11, negative control (WT plants). PCR amplification was performed using primers specific for *SpSOS1*, *SpAHA1,* or *hygB* gene (expected sizes of 980, 916, and 750 bp, respectively) with the corresponding DNA serving as the template. The PCR products were assessed by agarose gel electrophoresis. (TIF 835 kb)

Additional file 2: **Figure S2.** Expression of *SpSOS1* and *SpAHA1* genes in transgenic *Arabidopsis* lines. Total RNA was purified from leaves from the T3 generation of transgenic plants and used for RT-PCR analysis. The *Arabidopsis Actin* gene served as an internal control. (a) Expression of the *SpSOS1* gene in *SpSOS1-*transgenic plants was analyzed by RT-PCR. 1–12, different transgenic lines (lines 1–12). (b) Expression of *SpAHA1* gene in *SpAHA1-*transgenic plants as analyzed by RT-PCR. 1–11, different transgenic lines (lines 1–11). (c) Expression of *SpSOS1* and *SpAHA1* genes in *SpSOS1-SpAHA1* co*-*expressing plants as analyzed by RT-PCR. 1–10, different transgenic lines (lines 1–10). (TIF 531 kb)

Additional file 3: **Figure S3.** Na^+^ flux in roots of *Arabidopsis* plants grown in media without NaCl. Na^+^ flux in the roots of seven-day-old seedlings was measured using the NMT technique described in the Methods section. (a) Changes in the NMT signals are expressed as arbitrary units. (b) Na^+^ flux is expressed as the amount of efflux per second per square centimeter (pmol•cm^− 2^•s^− 1^). Data are presented as mean ± SE of three replicates. Same letter above the columns indicate that the differences at a *p* < 0.05 level among the different experimental cohorts are not significant statistically. SpSOS1, *SpSOS1*-overexpressing plants; SpAHA1, *SpAHA1*-overexpressing plants; SpSOS1-SpAHA1, *SpSOS1* and *SpAHA1* co-expressing plants; WT, wild-type plants. (TIF 456 kb)

Additional file 4: **Figure S4.** Schematic of T-DNA region in the binary vectors. (a) The pCAMBIA1300-*SpSOS1*, (b) T pCAMBIA1304-*SpSOS1*, (c) pCAMBIA1304-*SpSOS1*-*SpAHA1*, and (d) pCAMBIA1304-*SpAHA1* plasmids. (TIF 239 kb)

Additional file 5: **Table S1.** Sequences of primers used in this study. Small letters indicate restriction enzyme sites. (XLS 18 kb)

## Abbreviations

AHA: H^+^-ATPase
AVP/VP: Vacuolar H^+^-PPase
GUS: β-glucuronidase
HA: H^+^-ATPase
HO: Haem oxygenase
KOR: K^+^ outward rectifier
MDA: Malondialdehyde
MES: 2-(N-Morpholino) ethanesulfonic acid
MS: Murashige & Skoog
NHX: Na^+^/H^+^ antiporter
NSCC: Non-selective cation channels
PCR: Polymerase chain reaction
PM: Plasma membrane
RCD: Radical-induced cell death
RNAi: RNA interference
ROS: Reactive oxygen species
RT-PCR: Reverse transcription polymerase chain reaction
SOS: Salt overly sensitive
WT: Wild-type
