# Supplementary figures and images for: Co-expression of SpSOS1 and SpAHA1 in transgenic Arabidopsis plants improves salinity tolerance
Source: BMC Plant Biol. 2019 Feb 14;19:74. doi: 10.1186/s12870-019-1680-7 (PMC6376693; doi:10.1186/s12870-019-1680-7)

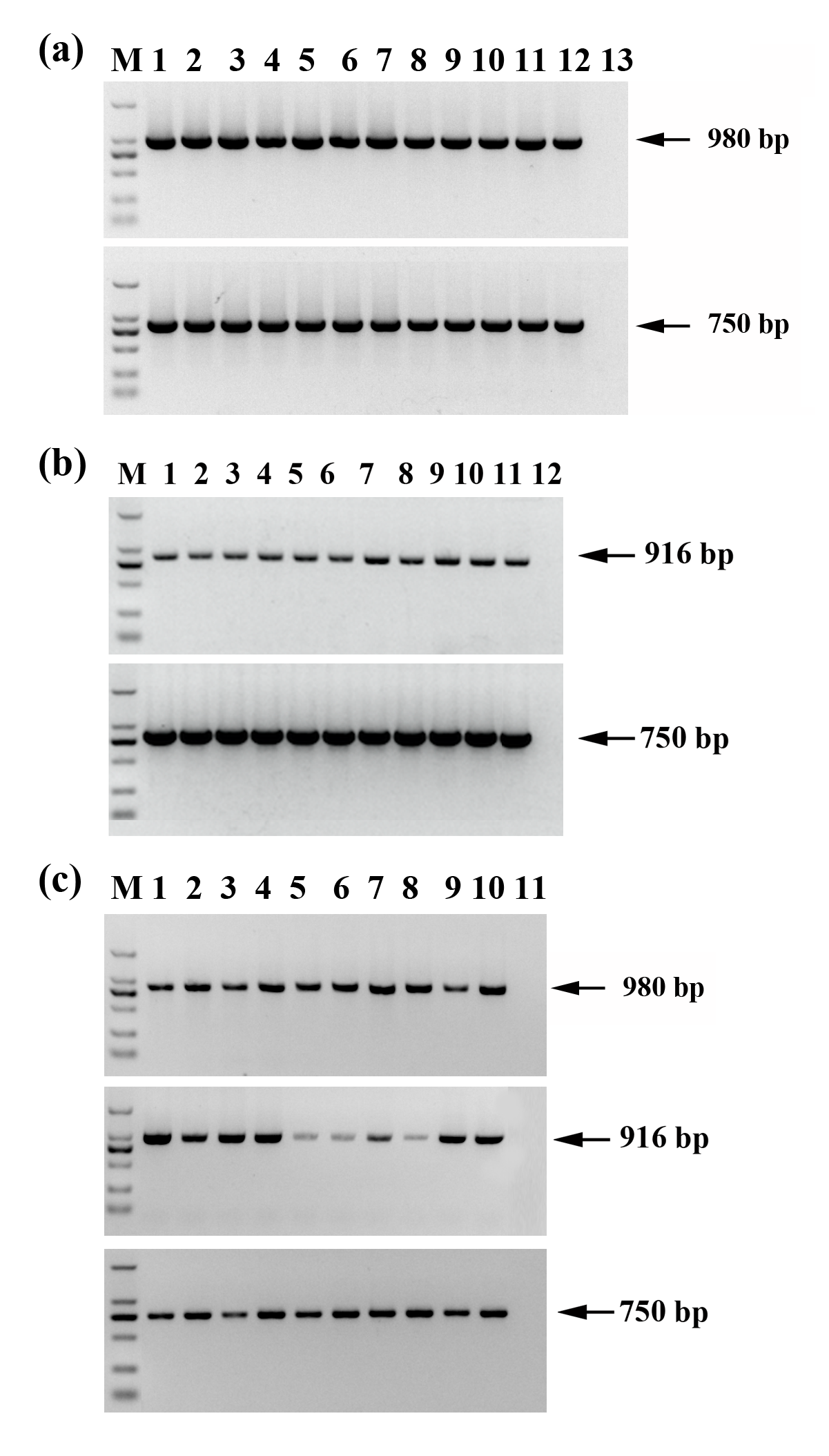

Supplement: Supplementary file 1 — Figure S1. Molecular identification of transgenic plants. DNA was purified from transgenic and WT plant leaves. (a) PCR identification of SpSOS1 transgenic plants. M: DL2000 marker (Sangon Biotech, China; No. B600335); 1–12, different transgenic lines (lines 1–12); 13, negative control (WT plants). (b) PCR identification of SpAHA1 transgenic plants. M: DL2000 marker; 1–11, different transgenic lines (lines 1–11); 12, negative control (WT plants). (c) PCR identification of SpSOS1 and SpAHA1 co-expressing plants. M: DL2000 marker; 1–10, different transgenic lines (lines 1–10); 11, negative control (WT plants). PCR amplification was performed using primers specific for SpSOS1, SpAHA1, or hygB gene (expected sizes of 980, 916, and 750 bp, respectively) with the corresponding DNA serving as the template. The PCR products were assessed by agarose gel electrophoresis. (TIF 835 kb) [file 12870_2019_1680_MOESM1_ESM.tif]

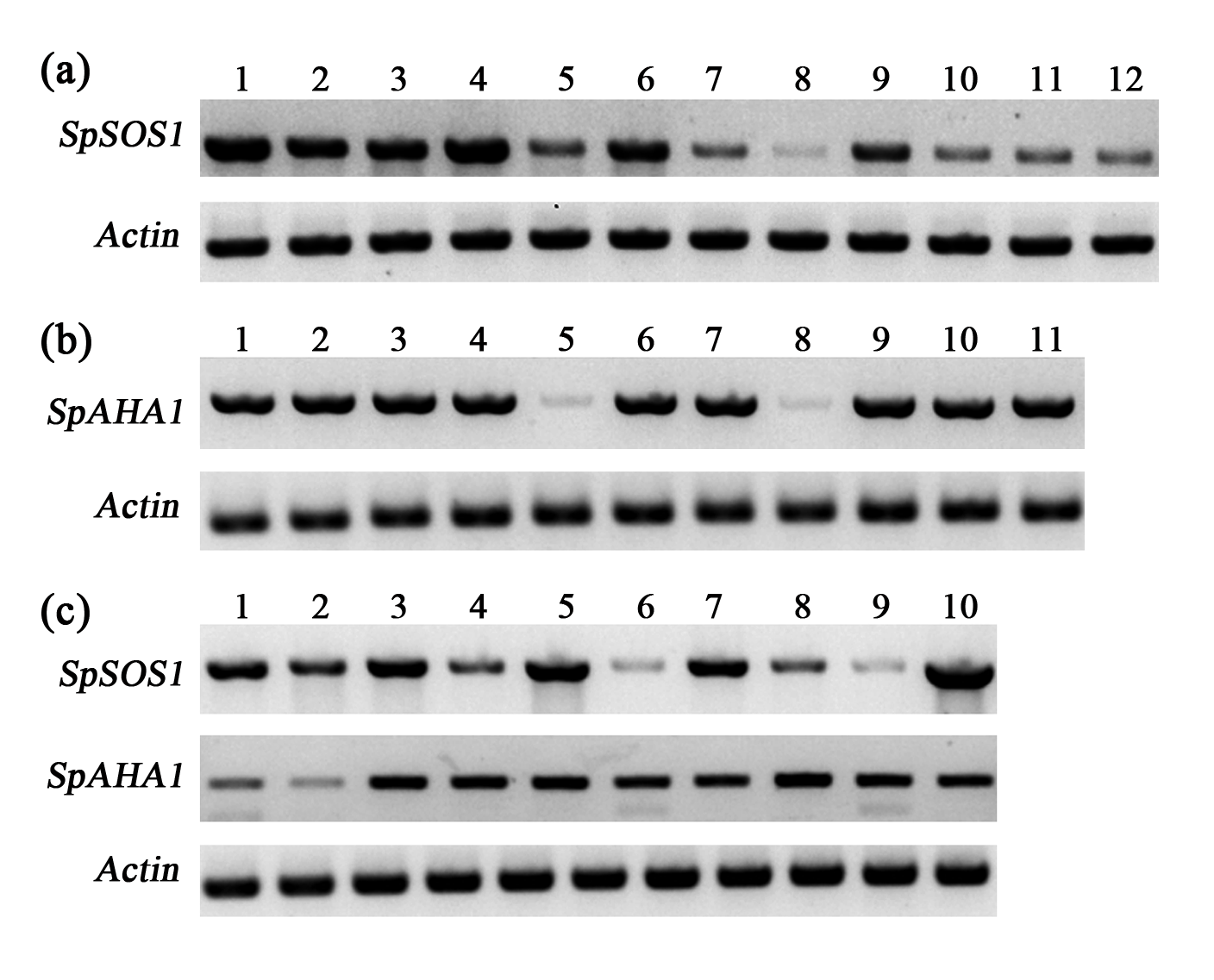

Supplement: Supplementary file 2 — Figure S2. Expression of SpSOS1 and SpAHA1 genes in transgenic Arabidopsis lines. Total RNA was purified from leaves from the T3 generation of transgenic plants and used for RT-PCR analysis. The Arabidopsis Actin gene served as an internal control. (a) Expression of the SpSOS1 gene in SpSOS1-transgenic plants was analyzed by RT-PCR. 1–12, different transgenic lines (lines 1–12). (b) Expression of SpAHA1 gene in SpAHA1-transgenic plants as analyzed by RT-PCR. 1–11, different transgenic lines (lines 1–11). (c) Expression of SpSOS1 and SpAHA1 genes in SpSOS1-SpAHA1 co-expressing plants as analyzed by RT-PCR. 1–10, different transgenic lines (lines 1–10). (TIF 531 kb) [file 12870_2019_1680_MOESM2_ESM.tif]

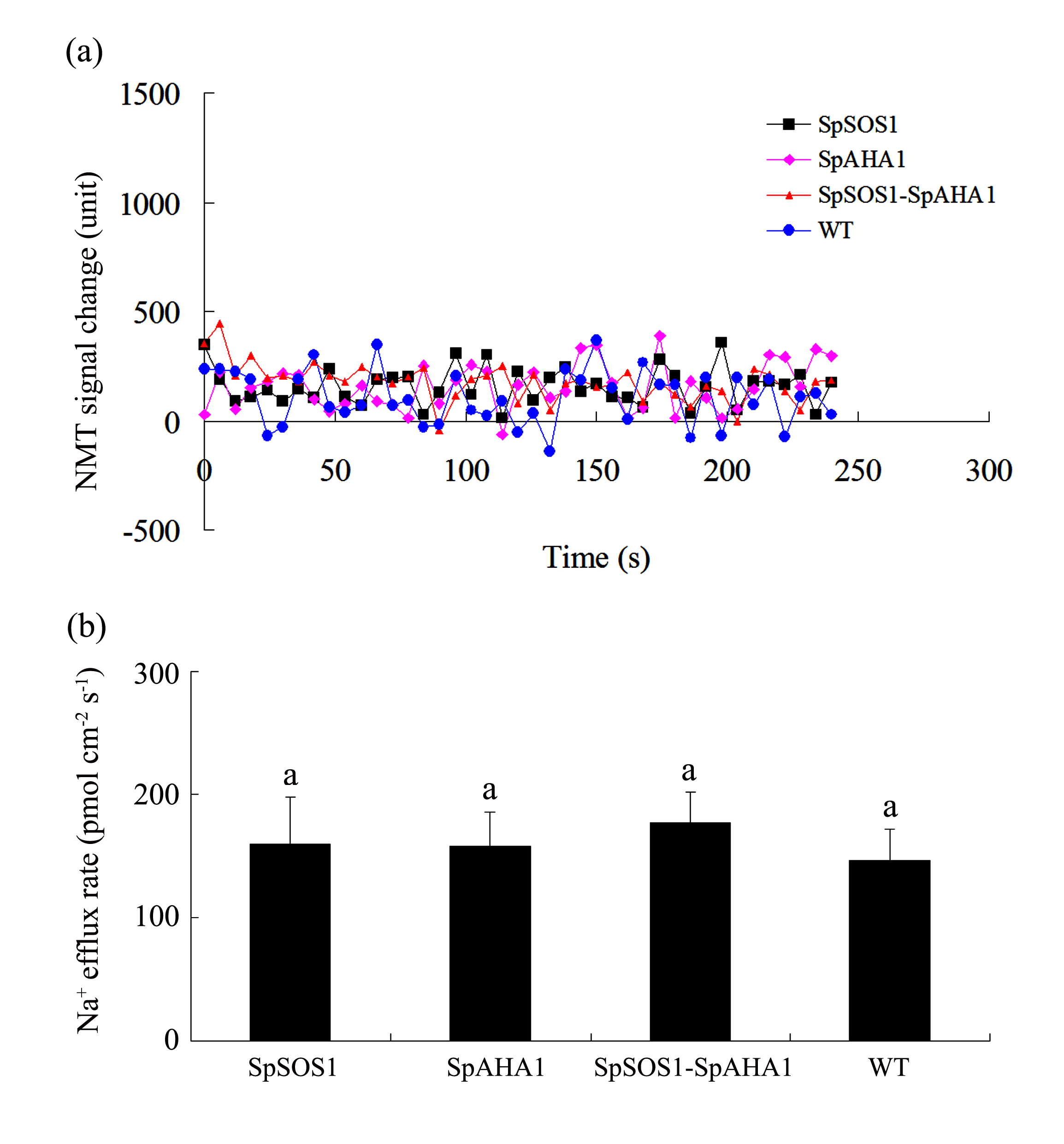

Supplement: Supplementary file 3 — Figure S3. Na+ flux in roots of Arabidopsis plants grown in media without NaCl. Na+ flux in the roots of seven-day-old seedlings was measured using the NMT technique described in the Methods section. (a) Changes in the NMT signals are expressed as arbitrary units. (b) Na+ flux is expressed as the amount of efflux per second per square centimeter (pmol•cm− 2•s− 1). Data are presented as mean ± SE of three replicates. Same letter above the columns indicate that the differences at a p < 0.05 level among the different experimental cohorts are not significant statistically. SpSOS1, SpSOS1-overexpressing plants; SpAHA1, SpAHA1-overexpressing plants; SpSOS1-SpAHA1, SpSOS1 and SpAHA1 co-expressing plants; WT, wild-type plants. (TIF 456 kb) [file 12870_2019_1680_MOESM3_ESM.tif]

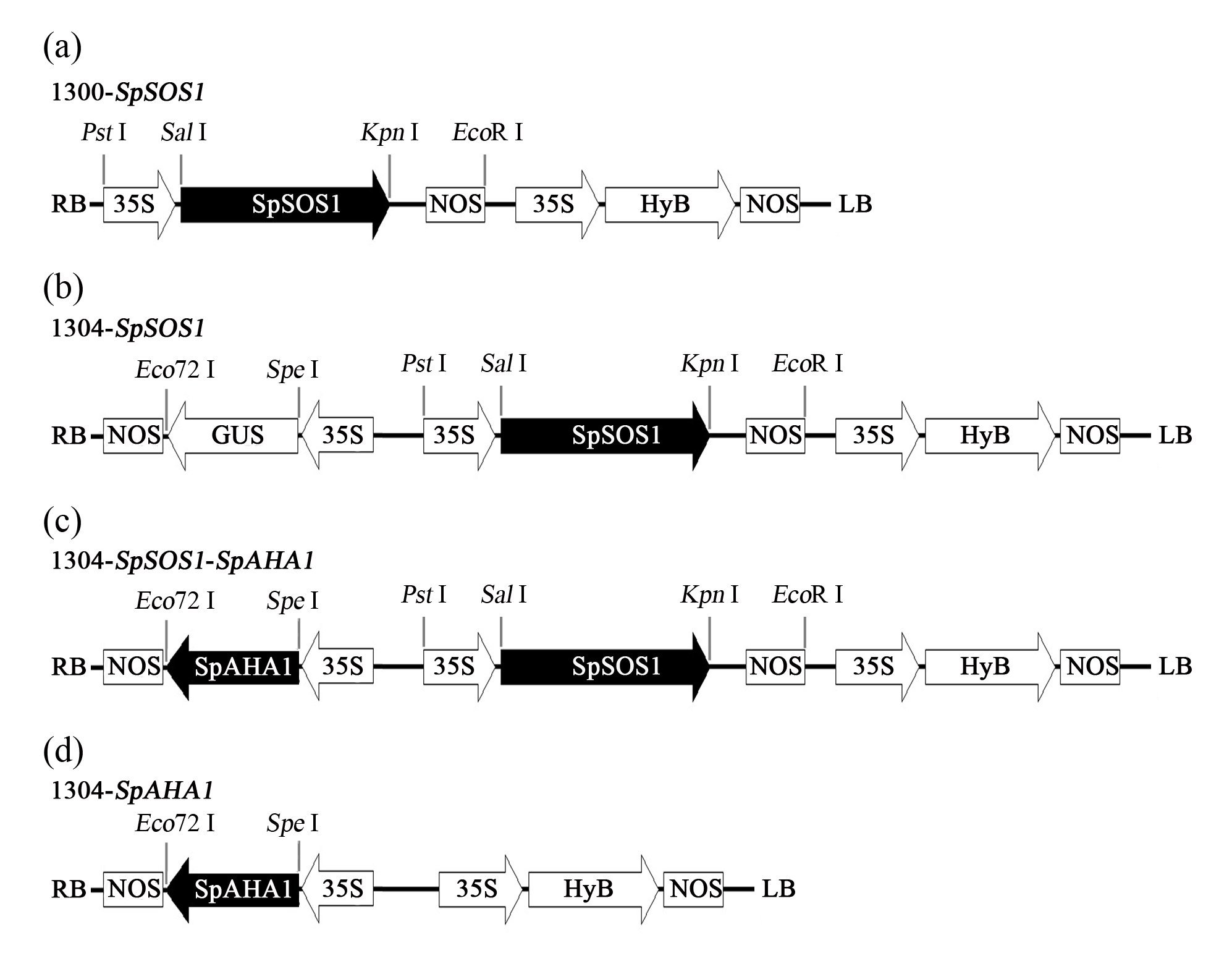

Supplement: Supplementary file 4 — Figure S4. Schematic of T-DNA region in the binary vectors. (a) The pCAMBIA1300-SpSOS1, (b) T pCAMBIA1304-SpSOS1, (c) pCAMBIA1304-SpSOS1-SpAHA1, and (d) pCAMBIA1304-SpAHA1 plasmids. (TIF 239 kb) [file 12870_2019_1680_MOESM4_ESM.tif]
